# Gene expression kinetics in Sepsis After Cardiac Surgery (SACS): a multicentric prospective observational study

**DOI:** 10.1186/s44158-025-00277-4

**Published:** 2025-09-26

**Authors:** Rosa Paola Radice, Giuseppe Martelli, Mauro D’Amora, Pierpaolo Dambruoso, Domenico Paparella, Raffaele Mandarano, Giuseppe Olivo, Massimo Scolaro, Domenico Sarubbi, Alessandro Strumia, Maria Calabrese, Andrea Scapigliati, Francesco Greco, Mary Nardi, Stefano Beccaria, Andrea Costamagna, Luca Brazzi, SIAARTI Study Group, Ezia Rotunno, Sergio Bevilacqua, Antonio Ferdinando Savino, Rosaria Vignale, Maria Enrica Antoniucci, Tommaso Pierani, Stefano Rizzo, Domenico Abelardo, Pasquale Raimondo, Gianluca Paternoster

**Affiliations:** 1 Department of Science, University of Basilicata, Via Dell’ateneo lucano 10, 85100 Potenza, PZ Italy; 2 AlgaeBioMed srl, via Luigi Kossuth 7, 00149 Rome, RM Italy; 3 AOR San Carlo - Via Potito Petrone, 85100 Potenza, PZ Italy; 4 Cardiovascular Anesthesia and Intensive Care Unit Ospedale Privato, Accreditato Santa Maria - GVM Care & Research, Bari, Italy; 5 Cardiac Anaesthesia and ICU, Department of Anaestesiology, Careggi University Hospital, Largo Giovanni Alessandro Brambilla, 3, 50134 Florence, FI Italy; 6 Unit of Cardiac Anesthesia and Intensive Care, Fondazione Toscana Gabriele Monasterio, Hospital of Massa, Pisa, Italy; 7 U.O.C. Di Anestesia E Rianimazione - Policlinico Campus Bio Medico Di Roma Scuola Di Specializzazione in Anestesia E Rianimazione, Università Campus Bio Medico Di Roma Via Alvaro del Portillo, 200 - 00128 Rome, Italy; 8 Department of Cardiovascular Sciences, Intensive Care Unit, Fondazione Policlinico Universitario “A. Gemelli” IRCCS, Rome, Italy; 9 Fondazione Di Religione E Di Culto, Casa Sollievo Della Sofferenza, San Giovanni Rotondo, FG Italy; 10 Cardiovascular Anaesthesia and Intensive Care, Mauriziano Hospital, Turin, Italy; 11 Dipartimento Di Scienze Chirurgiche, Università Degli Studi Di Torino, A.O.U Città della Salute e della Scienza di Torino, Turin, Italy; 12 Department of Medical and Surgical Sciences, Italy. Regional Epilepsy Centre, Magna Graecia University of Catanzaro, Great Metropolitan “Bianchi-Melacrino-Morelli” Hospital, Reggio Calabria, Italy; 13 Department of Precision-Regenerative Medicine and Jonic Area (DiMePRe-J), Section of Anesthesiology and Intensive Care Medicine, University of Bari “Aldo Moro”, Bari, Italy

**Keywords:** Septic shock, Cardiac Surgery, Gene expression, Interleukins, Cardiopulmonary bypass

## Abstract

**Objectives:**

Surviving Sepsis Campaign (SSC) defined Sepsis as “*life-threatening organ dysfunction caused by a dysregulated host response to infection*”. Sepsis remains one of the leading causes of morbidity and mortality (17-65 %) worldwide and it still remains a challenge to be defined and for which an appropriate treatment is desired. Different studies have been conducted on genes coding for inflammatory cytokines whose could predispose to the development of sepsis [e.g., IL-10 PD1 and WT1].

**Design:**

This multicentric observational prospective study aims to evaluate blinding the genetic expression kinetics of different molecules involved in the inflammatory process, IL10, PD1 and WT1, to search for a possible molecular predictive marker of sepsis.

**Setting:**

Nine University teaching Hospitals in Italy take part in this study in collaboration with the Department of Applied Science (DISBA) of the University of Basilicata.

**Participants:**

One hundred sixty-two patients, under elective cardiac and on pump surgery were enrolled in the study.

**Interventions:**

From each patient 4 blood samples were collected during and at the end of the surgery, following the study design.

**Measurements and main results:**

We observed, 30 minutes after the start of the surgery, lower gene expression levels of IL10 and PD1 in septic patients compared to non-septic (*p* <0.05), but considering all the timepoint there are differences in gene expression modulation between the groups.

**Conclusion:**

These results confirmed the dysregulated immune response in septic patients compared to non-septic, highlight how a measurement of the gene expression could help to optimize procedures and pay attention to more susceptible patients.

## Introduction

Sepsis remains one of the leading causes of morbidity and mortality worldwide [1, 2] , and despite the progress of medical science and research, it still remains a challenge to be defined and for which an appropriate treatment is desired [3, 4]. The incidence of post cardiac surgery sepsis appreciably varies in the literature: the incidence of classical defined sepsis was 0.39% according to Oliveira et al. [2], Michalopoulos et al. [5] report an incidence of 2% of severe sepsis after cardiac surgery, while Kollef et al. [6] found that 21.7% of cardiac surgery patient acquired at least one nosocomial infection postoperatively. Post-cardiac surgery sepsis presents high mortality (ranging from 17 to 65% of patients affected by sepsis) [2], increasing antibiotic resistance, prolonged need for intensive care unit (ICU) and hospital care and elevated costs. Sepsis is defined as a life-threatening organ disorder caused by a dysregulated host response to infection [1]. Septic shock is a subset of sepsis with hemodynamic implications and failure in cellular metabolism leading to organ disfunction. This is associated with higher mortality [7]. In sepsis, the immune response, activated by pathogen invasion, fails to restore homeostasis in the host, thus resulting in a pathological syndrome characterized by prolonged excessive inflammation and immunosuppression [8]. Cardiac surgery and in particular cardiopulmonary bypass (CPB) could be identified as entry routes for pathogens, these, can generate a systemic inflammatory response syndrome (SIRS). The main risk factors related to SIRS development are type of surgery, patient age, the presence of comorbidities (e.g., diabetes, rheumatoid arthritis, renal insufficiency), immunodeficiency, polytherapy, use of extracorporeal devices (e.g., catheters, drainage, mechanical ventilation), and genetic predisposition [9]. Others several factors, related to the use of CPB, contribute to the development of perioperative sepsis; these include blood contact with large artificial surfaces, the presence of drainage systems inside the bypass circuit for blood re-infusion, and hypothermia. All these factors are considered important SIRS triggers since they generate the release of endogenous inflammatory mediators [10, 11]. Sepsis is a complex biological interaction between host and pathogen; despite great researcher efforts, many aspects of the clinical picture, pathophysiology, and treatments remain challenging [12]. As a result, sepsis remains the leading cause of in hospital ICU admission, with high healthcare costs, furthermore it is a frequent cause of hospital readmission in sepsis survivors and has been reported as the last common path to death for infection [8]. For many years, the pathophysiology of sepsis has been focused around the aberrant host inflammatory response. It is now understood that the dysregulated response results from an imbalance between immune system and metabolic cells [13]. The initial stage of the host response to the pathogen is the activation of innate immune cells through the binding of pathogen-associated molecular patterns (PAMPs), such as bacterial endotoxins and fungal β-glucans to specific pattern recognition receptors (PRR) [14]; another source of such interaction are damage-associated molecular patterns (DAMPs) [15]. All these factors result in the activation of intracellular signal transduction pathways that induce the transcription and release of pro-inflammatory cytokines such as TNFα, IL-1 and IL-6.

### Genetic factors

Pro-inflammatory cytokines cause leukocyte activation and proliferation, complement system activation, upregulation of endothelial adhesion molecules, chemokine expression, and tissue factor production [10]. Among the factors predisposing to the development of sepsis, some genetic modifications, which do not yet have a clinical value, are found. Recently, the knowledge of the associations between genetic polymorphisms and sepsis has led to a better understanding of this syndrome ^4,^.^16^

For example, in the exon of the gene encoding lipopolysaccharide-binding protein (LPS), two Single Nucleotide Polymorphism (SNPs) have been identified at position 291(T/G) and 1306 (C/T), which cause a change in the amino acid sequence and lead to an increased susceptibility to sepsis [16].

Different studies have been conducted on genes coding for inflammatory cytokines whose modifications could genetically predispose to the development of sepsis:: IL-1α (-889 T/C), IL-1β (-511 C/T, + 3962 T /C), IL-1 RA (1100 T/C), IL-4 RA (+ 1902 G/A), IL-4 (-1098 T/G, -590 T/C, -33 T/C), IL-6 (-174 C/G, -560 G/A), IL-10 (-1082 A/G, -819 C/T, -592 C/A), IL-12 (-1188 C/A), γIFN (+ 874 A/T), TGF-β1 (codon 10 C/T, codon 25 G/C), TNF-α (-308 G/A, -238 G/A) and IL-2 (-330 T/G, + 166 G/T) [16]. The result of these studies demonstrated an association between alternations in genotype and allele frequency (e.g. IL-10 rs1800896 (-1082 A/G) and IL-6 rs1800795 (-174 G/C)) [17, 18]. Regarding interleukin-10, three SNPs localized in the IL-10 promoter region were identified: rs1800872 (-592 C/A), rs1800871 (-819 C/T) and rs1800896 (-1082 G/A). Among these, the polymorphism rs1800896 (-1082 A/G), where there is the substitution of an Adenine with a Guanine, is repeatedly associated with the development of sepsis and its mortality [4] due to its presence related to IL-10 mRNA expression, leading to an increased concentration of IL10 [19]. An excess of IL-10 induces immunoparalysis, exposing the individual carrying the G allele to secondary infections, resulting in sepsis and mortality with impaired bacterial clearance [20].

Other genes related with sepsis are the programmed cell death protein 1 (PD-1) and programmed death ligand 1 (PD-L1), and The *Wt1* (Wilms tumour) gene. PD1 represent important regulatory factors for the immune system as they can modulate the activity of T lymphocytes that encounter cells presenting the antigen (APCs). PD-1 expression levels are usually not detectable as they increase only following stimulation, followed by an increase in the number of T and B lymphocytes [21]. The expression of PD-1 reaches very high levels in T cells activated by the antigen and is reduced once the antigen is effectively eliminated [21]. Many publications report how to exist a correlation between sepsis and PD-1 expression dysregulation [22].

The *Wt1*(Wilms tumour) gene instead [23], is related with post-transcriptional and post-translational activity, and it also acts in the development of organs or tissues and in maintaining their homeostasis [24]. The different isoforms of the WT1 protein play a crucial role in the development of numerous organs, such as the kidneys, gonads, adrenal glands, heart and retina. WT1 is expressed in 1% of multipotent CD34 + stem cells in the bone marrow but also in the quiescent population of CD38- cells. Myeloid precursors express WT1 early, and this occurs in approximately 4% of common myeloid precursor cells (CMPs), in approximately 2% of granulocyte-monocyte precursor cells (GMPs), and approximately 17% of megakaryocytic erythroid precursor (MEPs) [23].

In the late stages of differentiation, WT1 expression is reduced, in fact only a small percentage of fully differentiated cells (< 1%) present level of WT1. The role of Wilms Tumor 1 (WT1) in sepsis is less well defined, although recent evidence suggests that WT1 is involved in hematopoietic differentiation, cytokine signaling, and immune cell regulation, making it a candidate of interest in the study of sepsis-related immune alterations.

The host immune response in sepsis is orchestrated by a network of cytokines, cell surface receptors, and transcription factors. Analysing the gene expression profiles of IL10, PD1, and WT1 in patients who develop sepsis after cardiac surgery may offer critical insights into the mechanisms driving immune dysregulation in this setting. Such analyses have the potential to identify early biomarkers of immune suppression, elucidate novel therapeutic targets, and contribute to the development of personalized immunomodulatory strategies aimed at restoring immune competence and improving patient outcomes. We will compare the genetic characteristics of two patients’ group: (1) who develop sepsis after cardiac surgery; (2) who not develop sepsis after cardiac surgery.

## Material and methods

### Study design

**Fig. 1:**
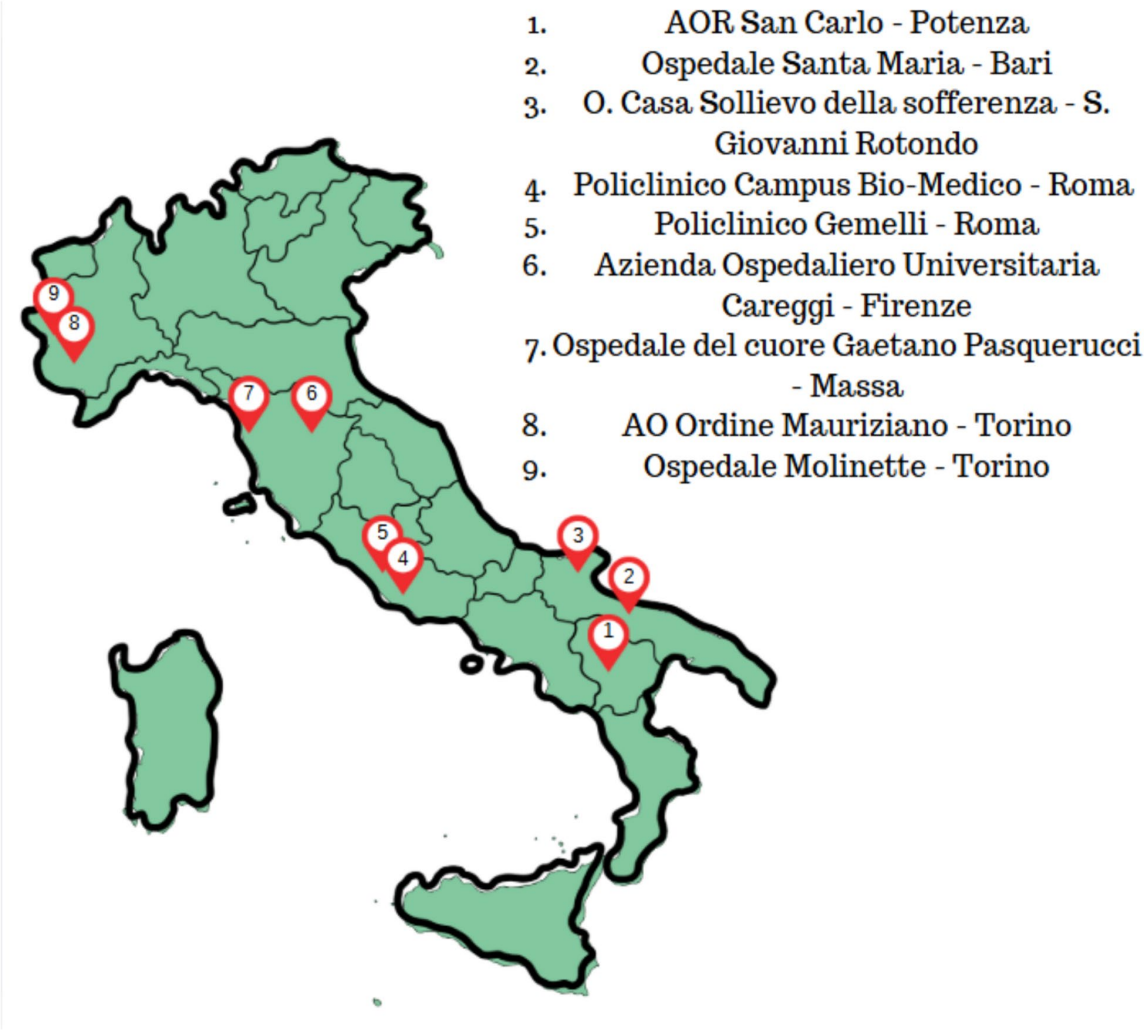
University teaching Hospital enrolled in the study

This is a prospective observational interventional study founded by Italian Society of Anaesthesia Intensive Care and Pain Therapy (SIAARTI). The study was conducted in nine University teaching Hospitals in Italy (Fig. 1). The study was approved by local ethical committee (Comitato Etico Unico Regionale per la Basilicata – Protocol number 20190050497). A total of 162 patients were enrolled in the study between October 2022 and January 2024.

Inclusion and exclusion criteria are reported in Table 1.. Sepsis was defined according to SSC criteria [1]. Patients included in the study were observed for 30 days after cardiac surgery. On the 30th day, the presence or absence of sepsis was assessed for all patients and reported in the Research Electronic Data Capture (RED cap). Four blood samples had been collected for each patient according to the following time points: (1) T0: Baseline (after the induction of anaesthesia and before surgical incision); (2) T1: During cardio-pulmonary bypass (Thirty minutes after the incitation of cardio-pulmonary bypass); (3) T2: After the end of the procedure (Just after the transfer from the operating theatre to the intensive care unit); (4) T3: After 24 h from the entry to the intensive care unit. Each sample and patient had a unique, encoded (anonym) identification code. All patients signed informed consent before the enrolment.

**Table 1.** Inclusion and exclusion criteria

**Inclusion criteria**	Elective cardiac surgery
	On pump surgery
	Age ≥18 years
**Exclusion criteria**	Emergency surgery
	Off pump surgery
	Active endocarditis
	Previous sepsis or septic shock
	Unsigned informed consent
	Age <18 years

### Data collection

Study data were collected and managed using REDCap electronic data capture tools hosted at SIAARTI Society [25, 26]. REDCap is a secure, web-based software platform designed to support data capture for research studies, providing 1) an intuitive interface for validated data capture; 2) audit trails for tracking data manipulation and export procedures; 3) automated export procedures for seamless data downloads to common statistical packages; and 4) procedures for data integration and interoperability with external sources.

### RNA extraction and qPCR

500 μl of whole blood are collected through heparin syringe and added to 500 μl of NucleoZOL Reagent (Macherey-Naghel). NucleoZOL reagent permits the RNA extraction from whole blood, specifically from nucleated cells without separate them. Each sample are storage at -20 °C following the manufacturing instruction. Once collected all the samples, 200 μl of DEPC water was added to all samples and vortexed. A treatment with DNase, to avoid DNA contamination, was performed before the next steps. The protocol used has been the one recommended by the manufacturer. RNA was quantified using the NanoDrop™1000 spectrophotometer (NanoDrop Technologies, Inc). RNA was retro-transcribed in cDNA using FIREScript® RT cDNA synthesis MIX (Solis Biodyne) and used at a final concentration of 10 ng. For qPCR assay, Power SYBR Green PCR Master Mix (Applied Biosystems®) was used following the standard procedure indicated by Applied Biosystem®. The amplification protocol was: 1) Holding Stage: 95 °C/10 min; 2) Cycling Stage: 40 cycles of 95 °C/15 s, 60 °C/1 min, 72 °C/ 1 min; 3) Melt Curve Stage: 95 °C/15 s; 60 °C/1 min; 95 °C/30 s; 60 °C/15 s. 7500 fast Real-Time PCR System (Applied Biosystems) and the 7500 Software v2.3 (Applied Biosystems) were employed. Real-time PCR primers are reported in Table 2. RNA extraction and gene analysis were conducted in a blind mode.

**Table 2:** Primer sequences used for qPCR

Primer	Forward sequence 5’-3’	Reverse sequence 5’-3’
IL-10	GGTTGCCAAGCCTTGTCTGA	CACATGCGCCTTGATGTCTG
PD1	CAGTTCCAAACCCTGGTGGT	GGCTCCTATTGTCCCTCGTG
WT1	GAGAGCCAGCCCGCTATTC	ATGAGTGGTTGGGGAACTGC
Actin	AGCGAGCATCCCCCAAAGTT	GGGCACGAAGGCTCATCATT

### Statistical analysis

Data were presented as mean ± standard deviation and as median and interquartile range for continuous variables, as appropriate. Categorical data were reported as percent frequency. Group comparisons were performed by the Student’s t test or Mann–Whitney U test for continuous variables, and the chi-square test (categorical data). Linear regression analysis was performed to test the relationship between IL10 expression and septic condition. The relative expression level of IL10, PD1 and Wt1 mRNA was normalized by the expression of housekeeping Actin mRNA in each sample. ΔCt (cycle threshold) values were obtained by: ΔCt = Ct target gene – Ct Actin.

The 2 − ΔΔCt method was used for data analysis [27]. Timepoint T0 was used as calibrator in ΔΔCt analysis to have a specific indication on the effect of surgical CPB.

## Results

### Study population

**Fig. 2:**
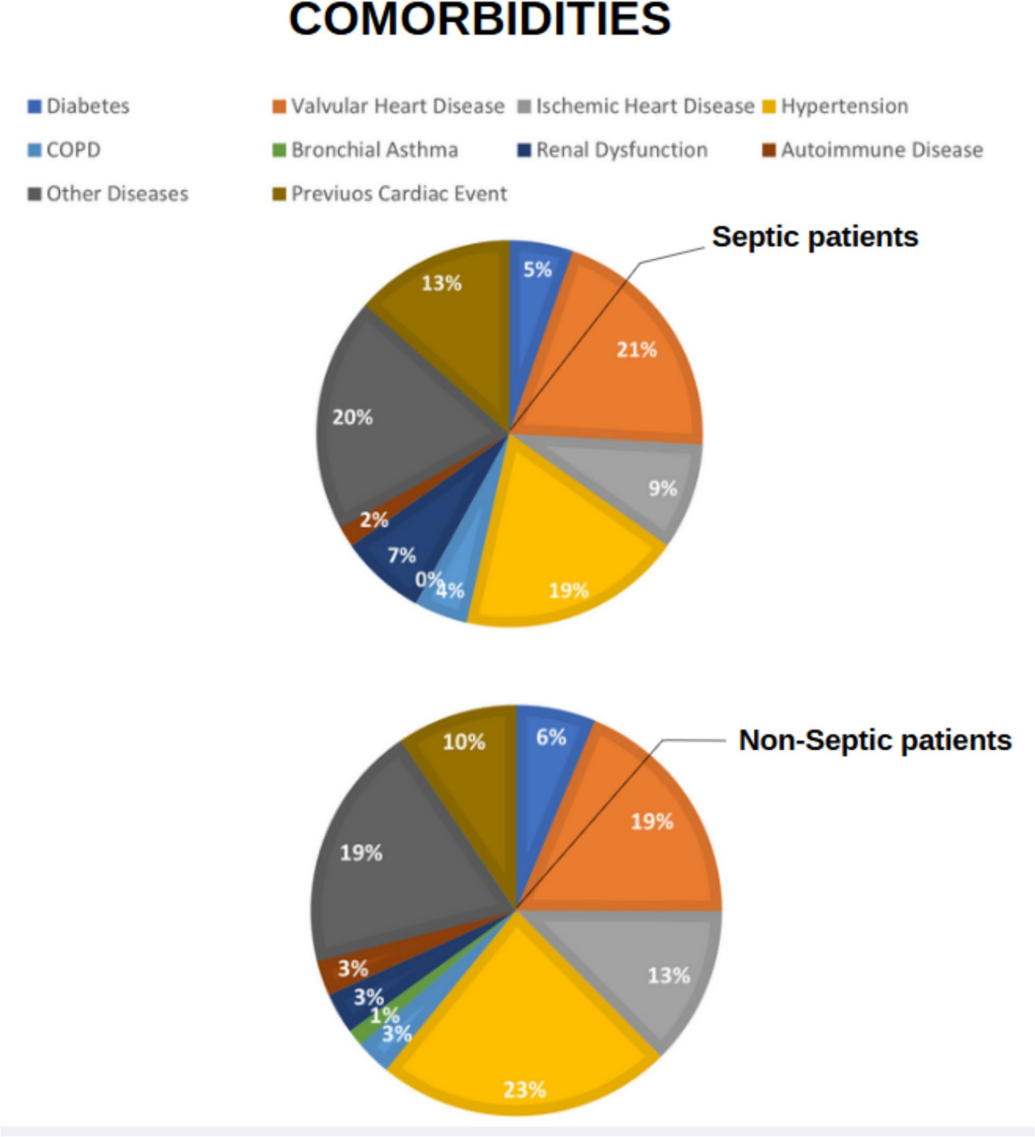
Comorbidities in Non-septic Group

One hundred sixty-two patients, the majority of whom were male (61.7%), have been enrolled in the study between October 2022 and January 2024. The study population characteristics are reported in Tables 3 and 4. Percentage of comorbidities in Septic and non-Septic patients are respectively reported in Fig. 2.

**Table 3:** Demographic and clinical data of the study population

Variables	Whole Group (*n* = 162)
Age (years)	68.6 ± 12.2
Gender, n. (%)	
Male	100 (61.7%)
Female	62 (38.3%)
Body Mass Index (kg/m^2^)	26.8 ± 5.1
Diabetes, n. (%), *N* = 161	34 (21.1)
Hypertension, n. (%), *N* = 161	129 (80.1)
COPD^a^, n. (%)	18 (11.1)
Bronchial asthma, n. (%)	6 (3.7)
CKD^b^, n. (%)	23 (14.2)
Valvular heart disease, n. (%), *N* = 161	110 (68.3)
Ischemic heart disease, n. (%)	68 (42)
Autoimmune diseases, n. (%)	15 (9.3)
Other comorbidities, n. (%)	112 (69.1)
Previous cardiac event, n. (%), *N* = 161	
No	102 (63.3)
AMI^c^	31 (19.3)
Other	28 (17.4)
Surgical access, n. (%), *N* = 161	
Sternotomy	106 (65.8)
Mini sternotomy	25 (15.5)
Right thoracotomy	2 (1.3)
Right mini thoracotomy	28 (17.4)
Clamping, n. (%), *N* = 160	
No	4 (2.5)
Direct clamping	154 (95.1)
Endoaortic	2 (1.2)
CBP^d^ (mins), *N* = 153	98 (70–37)
Aortic cross clamping time (mins), *N* = 154	72 (50–102)

^a^*COPD* Chronic obstructive pulmonary disease

^b^*CKD* Chronic kidney disease

^c^*AMI* Acute myocardial infarction

^d^*CPB* Cardiopulmonary bypass; *N* = refers to the total number of patients for whom information is available

**Table 4:** Comparison between septic and non-septic patients

Variables	Non-septic (*n* = 137)	Septic (*n* = 25)	*p*
Age (years)	68.5 ± 11.9	68.8 ± 13.9	0.925
Gender, n. (%)			0.847
Male	85 (62)	15 (60)	
Female	52 (38)	10 (40)	
Body Mass Index (kg/m^2^)	26.1 ± 3.7	26.9 ± 5.3	0.335
Diabetes, n. (%), *N* = 161	28 (20.6)	6 (24.0)	0.701
Hypertension, n. (%), *N* = 161	108 (79.4)	21 (84)	0.597
COPD^a^, n. (%)	13 (9.5)	5 (20.0)	0.124
Bronchial asthma, n. (%)	6 (4.4)	0 (0)	0.286
CKD^b^, n. (%)	15 (10.9)	8 (32.0)	**0.006**
Valvular heart disease, n. (%), *N* = 161	87 (64)	23 (92)	**0.006**
Ischemic heart disease, n. (%)	58 (42.3)	10 (40.0)	0.828
Autoimmune diseases, n. (%)	13 (9.5)	2 (8.0)	0.813
Other comorbidities, n. (%)	90 (66.2)	22 (88.0)	**0.029**
Previous cardiac event, n. (%), *N* = 161			**0.031**
No	92 (67.6)	10 (40.0)	
AMI^c^	23 (16.9)	8 (32.0)	
Other	21 (15.4)	7 (28.0)	
Surgical access, n. (%), *N* = 161			0.392
Sternotomy	92 (67.6)	14 (56.0)	
Mini sternotomy	21 (15.4)	4 (16.0)	
Right thoracotomy	1 (0.7)	1 (4.0)	
Right mini thoracotomy	22 (16.2)	6 (24.0)	
Clamping, n. (%), *N* = 160			0.135
No	2 (1.5)	2 (8.0)	
Direct clamping	131 (97.0)	23 (92.0)	
Endoaortic	2 (1.5)	0 (0)	
CBP^d^ (mins), *N* = 153	95 (67–133)	122 (90–186)	**0.032**
Aortic cross clamping time (mins), *N* = 154	70 (50–98)	75 (55–118)	0.256

^a^COPD: chronic obstructive pulmonary disease

^b^CKD: chronic kidney disease

^c^AMI: acute myocardial infarction

^d^CBP: cardiopulmonary bypass; *N* = refers to the total number of patients for whom information is available

The incidence of sepsis at 30 days following Cardiac Surgery was 15.43% (25 patients). These results are markedly different if compared to the incidence of sepsis reported in literature.

As regards cardiac surgery, we found significant difference between the two groups in CBP time: 122 min (90–186) in septic patients vs 95 min (67–133) in non-septic patients (*p* < 0.032). No statistical difference was observed in aortic cross clamping time (*p* = 0.256). In terms of surgical access, 65% of patients underwent sternotomy, 15% mini-sternotomy, 1% right thoracotomy, and 17% right mini-thoracotomy. As for the type of clamping, 95% of patients received direct aortic cross clamping, 1% endo-clamping, and 2% did not undergo aortic cross clamping. No statistical significances were observed in terms of surgical access and type of aortic cross clamping between the two groups (*p* > 0.05). We also found a significant difference in the prevalence of valvular heart disease, which was higher in the septic patients (92%) compared to in the non-septic patients (64%, *p* < 0.006). This association was confirmed by logistic regression analysis. The crude model revealed that patients having valvular heart disease had an odds ratio for sepsis approximately sixfold higher than the non-septic patients (OR: 6.5; 95%CI: 1.5–28.6; *p* = 0.014). After adjusting for traditional confounder (age, gender, and BMI), this association still maintained significance (OR: 7.1; 95%CI: 1.5–32.2; *p* = 0.012). Furthermore, we observed a greater prevalence of CKD in the septic patients (32%) than the non-septic ones (11%, *p* = 0.006). In the unadjusted model, patients with CKD had a higher risk to develop sepsis compared to those without (OR: 3.8; 95%CI: 1.4–10.4; *p* = 0.008). The association retained statistical significance even adjusted for traditional confounder (OR: 5.1; 95%CI: 1.6–16.0; *p* = 0.005).

### Genetic analysis

**Fig. 3:**
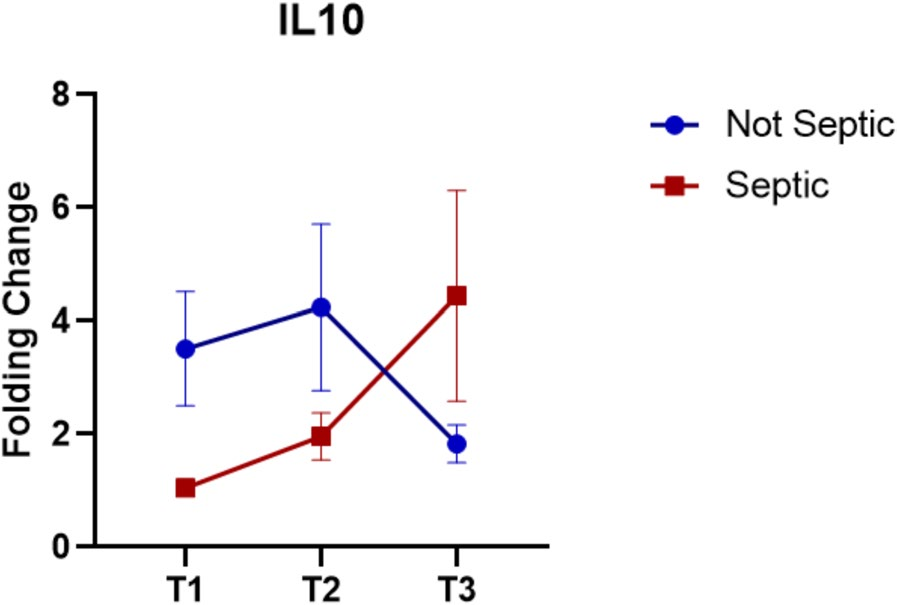
IL-10 expression. Data show as FC ± SEM

The genetic expressions analysis, show how the IL10 expression is markedly different between septic and non-septic patients (Fig. 3). The data highlight that 30 min after the start of surgery (time point 1, T1), septic patients had much lower levels of IL-10 expression compared to non-septic patients (FC = 1.05 Vs. 3.51, *p* > 0.05). At T3, septic patients have high levels of IL10 expression (FC = 4.44), compared to non-septic patients (FC = 1.83) (*p* < 0.05).

**Fig. 4:**
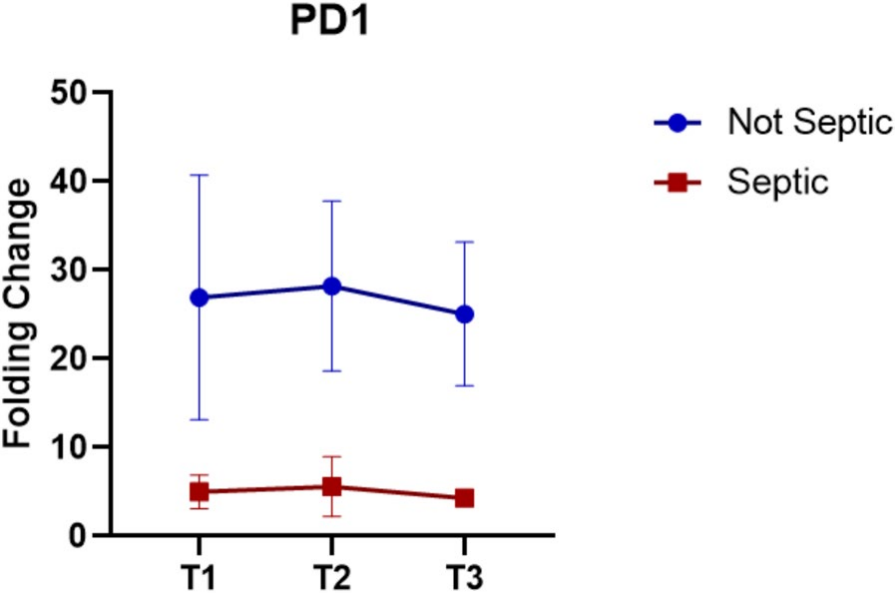
PD1 expression. Data show as FC ± SEM

The expression of PD1 (Fig. 4), an immunoregulatory gene in septic patients appears to be totally deactivated, unlike non-septic patients, which instead show higher values and modulate its expression. In detail, at T1, PD1 level in non-septic patients are higher (FC = 26.93) compared with septic patient (FC = 5.00) (*p* < 0.05). After the end of the procedure (T2) we observed a slight, although not significant compared with T1, increase in PD1 expression in both groups (FC Non septic = 28.23 Vs FC Septic = 5.61). At T3, the values had normalized showing no statistical difference within the group (*p* > 0.05), but a significant difference between the groups (FC Non septic = 25.08 Vs FC Septic = 4.33) (*p* < 0.05).

**Fig. 5:**
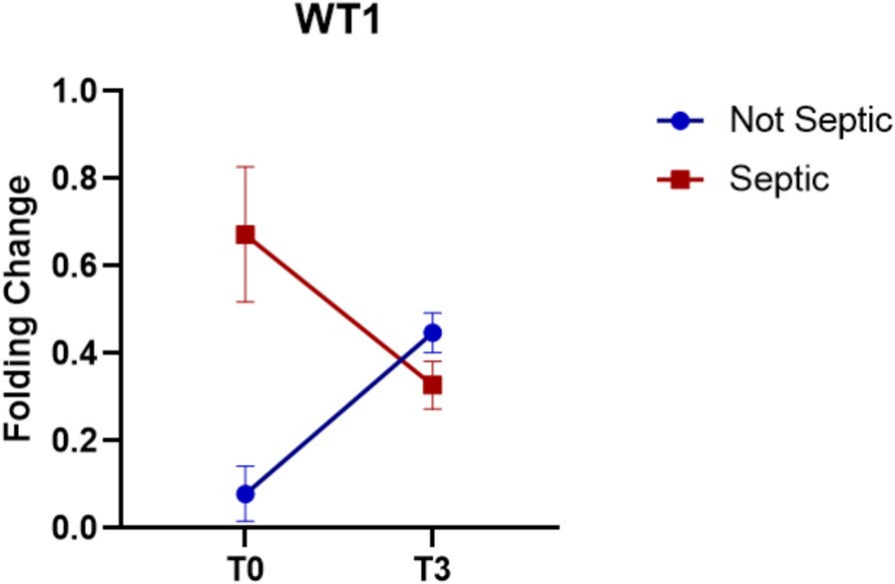
WT1 Expression. Data show as FC ± SEM

However, the results obtained from the analysis of WT1 showed an interesting trend (Fig. 5); however, it is evident that in patients who developed sepsis, WT1 was over-expressed compared to patients who did not develop sepsis (FC = 0.67 vs FC = 0.079, respectively) (*p* < 0.05).

In a linear regression model having the natural logarithm (LN) delta (∆) of ∆∆CT (LN ∆∆CT at T2 – LN ∆∆CT at T0) as a dependent variable, septic condition is directly associated to a major ∆ between T0 and T2 with a *p* value that is at threshold of statistical significance (*p* = 0.078, standardized regression coefficient β = 0.111, 95%CI: -0.06 – 1.14), probably due to sample size. This analysis was adjusted for baseline value of ∆∆CT since it differs between septic/non septic patients (Table 5). In addition, this model revealed that the baseline value is inversely associated to ∆ i.e., the higher baseline value, the lower the ∆ between T0 and T2. Finally, this result suggests a trend worthy of further analysis.

**Table 5:** Statistical model used for the genetic analysis

Variables	Regression coefficients β	95% CI^2^	*p*
Septic condition (0 = no; 1 = yes)	0.111	-0.06, 1.14	0.078
LN_∆∆CT_T0	-0.722	-0.98, -0.70	< 0.001

## Discussion

The study population show how the incidence of sepsis is different if compared with data reported in literature. We carefully analyzed this gap, and we think is due to a different studied population (2010 vs 2024). The patient’s age, subjected at CBP in 2024, is higher than in 2010. It must also be considered that the initial conditions of the patients greatly affect the results of the analysis. The presence of valvular heart disease has been shown to be an important trigger contributing to the development of sepsis, representing a significant risk factor. Patients with valvular heart disease (VHD) undergoing cardiac surgery with cardiopulmonary bypass (CPB) are at increased risk of developing sepsis in the postoperative period. This vulnerability is multifactorial, involving the combined effects of structural valve pathology, immune perturbation, surgical trauma, and CPB-induced systemic inflammation. This is justified for several reasons. Patients who receive valve repair are usually subjected to a longer CBP time which has been found to be significantly higher in septic patients [28]. Furthermore, they have a greater susceptibility to develop myocardial infections which can generate a systemic infection [29].. CDKs are also important risk factors, as demonstrated by the results obtained. CKD is characterized by a multifactorial impairment of the immune system, predisposing patients to develop systemic infections. At the immunological level, CKD induces immune dysregulation. On the one hand, the uremic environment is associated with chronic low-grade inflammation, which is reflected in elevated levels of circulating cytokines such as IL-6, TNF-α, and C-reactive protein [30, 31]. On the other hand, there is substantial evidence of innate and adaptive immune dysfunction, including impaired neutrophil chemotaxis and phagocytosis [32], reduced monocyte antigen presentation [33], T-cell exhaustion [34], and reduced B-cell reactivity [35]. These abnormalities are exacerbated by the accumulation of uremic toxins, such as indoxyl sulfate and p-cresyl sulfate, which interfere with immune cell signaling and antimicrobial defenses [36]. Furthermore, patients with CKD commonly suffer from intestinal dysbiosis, resulting in increased intestinal permeability. This facilitates the entry of microbial products such as lipopolysaccharide into the circulatory system [37, 38]. Importantly, patients with advanced chronic kidney disease (CKD) are subject to repeated healthcare exposures, including vascular access, indwelling catheters, and frequent hospital admissions, all of which increase the risk of pathogen acquisition and nosocomial sepsis [39]. From a cardiovascular perspective, the interaction between CKD and sepsis has direct relevance as it represents an immunodeficiency and pro-inflammatory state that significantly alters the trajectory of infectious diseases. The results of the study population demonstrate how there is a characterizing basis for each individual patient. For this reason, during the genetic analyses, the T0 point was used to normalize the initial condition of each subject and thus eliminate the influences that these could have on the analysis. The objective was precisely to evaluate how only the operative triggers could modulate gene expression.

All the results explain the relationship between dysregulation of the immune system and sepsis. Interleukin-10 (IL-10) is a key anti-inflammatory cytokine that plays a regulatory role in sepsis by limiting the host immune response to infection. The results obtained show in fact how patients who have not developed sepsis start from an initial advantageous condition compared to patients who develop sepsis, who show very low expression levels at T1. At T3, however, the results show how septic patients have exponentially increased the expression of IL10. This makes it clear how the immune system is dysregulated and appears to be blocked and unable to respond to the inflammatory stimuli. During sepsis, IL-10 is rapidly upregulated in response to pro-inflammatory cytokines such as TNF-α and IL-6. Its primary function is to suppress excessive inflammation, preventing tissue damage by inhibiting the production of pro-inflammatory mediators, reducing antigen presentation, and downregulating macrophage and dendritic cell activity. However, in the context of severe or late-stage sepsis, persistently high levels of IL-10 can contribute to immunosuppression, increasing the risk of secondary infections and poor outcomes [40, 41].

As we describe before, PD1 is involved differently in immunoregulations; one of this difference is the antigen presentation. Zhang et al. [22] describe how PD1 works during this important step of the immune response. T cells interact with APC cells during antigen presentation through different pathways. Usually, the binding between PD1 present on the T cell and its ligand, PD-L1, limits the activation of the T cell by inhibiting the protein kinase B (AKT) signaling pathway. PD1 uses the help of Src homology region 2 domain-containing phosphatases (SHP2) to also regulate the zeta chain of T cell receptor-associated protein kinase 70 (Zap70) and phosphoinositide 3 kinase (PI3K). However, in the present study, conducted on a much larger patient cohort than those reported in the literature, we demonstrate how PD1 is downregulated in patients who developed sepsis. Furthermore, it is well known in biological processes that binding of PD1 to its ligand occurs not only on T cells but also on B cells of the immune system. The prolonged interaction between regulatory B cells (Bregs) and T cells reduces subsequent contacts between T cells and dendritic cells, thereby hindering the antigen presentation process and subsequent T cell activation. Additionally, Bregs modulate humoral immunity by influencing the activity of follicular helper T cells, which play a role in differentiating B cells into antibody-producing plasma cells. This effect is mediated by the high expression of programmed death-ligand 1 (PD-L1) on Bregs, which binds to PD-1 on T cells. The PD-1/PD-L1 interaction inhibits the functionality and proliferation of effector T cells. Furthermore, Bregs exert suppressive effects by releasing elevated levels of IL-10, which hinders T cell differentiation [42]. The link between IL10 and PD1 was evident. In our septic patient cohort, low initial IL10 levels impaired the capacity to initiate an effective immune response. In contrast, surgical intervention acted as a strong inflammatory trigger, leading to subsequent IL10 overproduction. At the same time, low PD1 levels compromised T cell regulation impairing their ability to modulate B cell-mediated antibody production. As a result, B cells were not properly activated, and the pro-inflammatory process required to counteract systemic infection did not occur.

These results are precisely interconnected with the results obtained regarding the expression of WT1. The WT1 expression levels in the patients before surgery, given the nature of the WT1 gene [43], should be very low, but the results show how septic patients have a high expression level of WT1 at time T0.

It is important to underline that in the present study, the control cohort is represented by sick patients requiring surgery who did not subsequently develop sepsis. This clarification is necessary as it corroborates the results obtained in the two groups' comparison. It is possible that WT1, being involved in cellular differentiation at the bone marrow level, is responsible for hyperstimulation of the immune response; this has generated a high number of cells of the immune system which, in a condition of dysregulation, have not been able to react to pathogenic stimuli. Condition absents in the patients of the control cohort, who in a condition of normal expression of WT1, saw an increase (*p* < 0.05) only after the surgery (FC = 0.44), demonstrating an effective stimulation of the cells of the immune system, which were able to react to the possible pathogen attack.

These preliminary data suggest a different genetic profile in septic patients that could open future trends in research to evaluate the genetic profile, prior to surgery, to possibly identify patients at risk of sepsis.

## Conclusion

This study highlights a significantly higher incidence of sepsis (15.43%) following cardiac surgery with cardiopulmonary bypass (CPB) compared to previously reported literature, suggesting a shift potentially attributable to the evolving demographic and clinical characteristics of surgical candidates. The analysis identified valvular heart disease and chronic kidney disease (CKD) as major independent risk factors for postoperative sepsis, with adjusted odds ratios of 7.1 and 5.1, respectively. These conditions likely contribute to immune dysregulation through prolonged surgical exposure, systemic inflammation, and pre-existing immunosuppressive states.

Genetic analyses further support the hypothesis of an immunological basis for sepsis susceptibility. Septic patients showed distinct expression patterns of key immunomodulatory genes. Notably, IL-10 expression was initially suppressed and later elevated, indicative of a delayed and dysregulated anti-inflammatory response. PD-1, a central immune checkpoint molecule, was consistently downregulated in septic patients, possibly contributing to impaired T-cell regulation and insufficient antigen presentation. WT1 expression, elevated at baseline in septic patients, suggests an aberrant priming of the immune system prior to surgery, possibly reflective of underlying immune hyperreactivity that becomes maladaptive in the perioperative context.

Despite these insights, several limitations must be acknowledged. The single-center design and modest sample size, particularly within the septic subgroup, may limit generalizability and the statistical power of certain findings. Furthermore, while the observational nature of the study precludes causal inference, it does underscore important associations warranting further investigation.

Looking forward, these findings suggest that preoperative genetic and immunological profiling could help identify patients at elevated risk for postoperative sepsis, enabling personalized perioperative management strategies. Larger multicenter studies and mechanistic research into immune-genetic interactions in surgical patients are needed to validate these preliminary findings and refine predictive models of sepsis risk. Ultimately, such approaches may enhance patient stratification and inform targeted interventions to mitigate the burden of sepsis in cardiac surgery.

## Data Availability

No datasets were generated or analysed during the current study.

## Declarations

Ethics approval and consent to participate.

The study is approved by CEUR Basilicata with protocol number 20190050497. All the patients enrolled in the study have signed the consent declaration as inclusion criteria.

## References

[1] De Backer D, Deutschman CS, Hellman J, et alCrit Care Med Lippincott Williams and Wilkins,

[2] De Oliveira DC, De Oliveira Filho JB, Ferreira Silva R, et al: Sepsis in the postoperative period of cardiac surgery: problem description. Arq Bras Cardiol Sociedade Brasileira de Cardiologia - SBC, 94:352–6, 2010. Retrieved from: https://www.scielo.br/j/abc/a/RkVJSGSj9jPJkKXs7L5nYwn/?lang=en. Cited 2024 10.1590/s0066-782x2010000300012 20730262

[3] Chiu C, Legrand M (2021) Epidemiology of sepsis and septic shock. Curr Opin Anaesthesiol 34(2):71–6. 10.1097/ACO.0000000000000958 33492864 10.1097/ACO.0000000000000958

[4] Gupta DL, Sinha T, Bhoi S, et al: Cytokine Gene Polymorphism and Sepsis.Infectious Process and Sepsis IntechOpen,2020. Retrieved from: https://www.intechopen.com/state.item.id. Cited 2022

[5] Michalopoulos A, Stavridis G, Geroulanos S. Severe sepsis in cardiac surgical patients. Retrieved from: https://academic.oup.com/ejs/article/164/3/217/6041549. Cited 2024] 10.1080/110241598750004670 9562283

[6] Kollef MH, Sharpless L, Vlasnik J et al (1997) The impact of nosocomial infections on patient outcomes following cardiac surgery. Chest Elsevier 112:666–675 10.1378/chest.112.3.666 9315799

[7] Seymour CW, Liu VX, Iwashyna TJ, et al (2016) Assessment of clinical criteria for sepsis. JAMA Am Med Assoc 315:762. Retrieved from: http://jama.jamanetwork.com/article.aspx?doi=10.1001/jama.2016.0288. Cited 2023 10.1001/jama.2016.0288 PMC5433435 26903335

[8] van der Poll T, van de Veerdonk FL, Scicluna BP, et al (2017) The immunopathology of sepsis and potential therapeutic targets. Nat Rev Immunol 17:407–20. Retrieved from: https://pubmed.ncbi.nlm.nih.gov/28436424/. Cited 2022 10.1038/nri.2017.36 28436424

[9] Keegan J, Wira CR (2014) Early identification and management of patients with severe sepsis and septic shock in the emergency department. Emerg Med Clin North Am W.B. Saunders 32:759–76 10.1016/j.emc.2014.07.002 25441033

[10] Day JRS, Taylor KM (2005) The systemic inflammatory response syndrome and cardiopulmonary bypass. Int J Surg Elsevier 3:129–140 10.1016/j.ijsu.2005.04.002 17462274

[11] Innelli P, Lopizzo T, Paternò G, et al (2023) Dipeptidyl Amino-Peptidase 3 (DPP3) as an early marker of severity in a patient population with cardiogenic shock. Diagnostics 2023, Vol. 13, Page 1350 Multidisciplinary Digital Publishing Institute, 13:1350. Retrieved from: https://www.mdpi.com/2075-4418/13/7/1350/htm. Cited 2024 10.3390/diagnostics13071350 PMC10093224 37046568

[12] Paternoster G, Guarracino F (2016) Sepsis after cardiac surgery: from pathophysiology to management. J Cardiothorac Vasc Anesth. Elsevier 30:773–80. Retrieved from: http://www.jcvaonline.com/article/S1053077015009398/fulltext. Cited 2022 10.1053/j.jvca.2015.11.009 26947713

[13] Genga KR, Russell JA (2017) Update of sepsis in the intensive care unit. J Innate Immun Karger Publishers 9:441–55. Retrieved from: https://www.karger.com/Article/FullText/477419. Cited 2022 10.1159/000477419 PMC6738902 28697503

[14] van der Poll T, Opal SM (2008) Host-pathogen interactions in sepsis. 8. Retrieved from: http://infection.thelancet.com. Cited 2022 10.1016/S1473-3099(07)70265-7 18063412

[15] Kawai T, Akira S (2010) The role of pattern-recognition receptors in innate immunity: update on Toll-like receptors. Nat Immunol. 10.1038/ni.1863 20404851 10.1038/ni.1863

[16] Walley KR, Boyd JH (2020) Lipoprotein biology in sepsis. Crit Care Med Lippincott Williams and Wilkins 48:1547–1549 10.1097/CCM.0000000000004519 32925267

[17] Gupta DL, Nagar PK, Kamal VK, et al (2015) Clinical relevance of single nucleotide polymorphisms within the 13 cytokine genes in North Indian trauma hemorrhagic shock patients. Scand J Trauma Resusc Emerg Med 23. Retrieved from: https://pubmed.ncbi.nlm.nih.gov/26561011/. Cited 2022 10.1186/s13049-015-0174-3 PMC4642631 26561011

[18] Zhang Y, Cui X, Ning L, et al. (2017) The effects of tumor necrosis factor-α (TNF-α) rs1800629 and rs361525 polymorphisms on sepsis risk. Oncotarget Impact J LLC 8:111456. Retrieved from: /pmc/articles/PMC5762335/. Cited 2022 10.18632/oncotarget.22824 PMC5762335 29340067

[19] Zhang N, Wang S, Fan Y, et al (2022) Association between IL10 polymorphisms and the susceptibility to sepsis: a meta-analysis. Biochem Genet Springer 1–14. Retrieved from: https://link.springer.com/article/10.1007/s10528-022-10310-8. Cited 2023 10.1007/s10528-022-10310-8 36534332

[20] Kang X, Kim H-J, Ramirez M et al (2010) The septic shock-associated IL-10 −1082 A > G polymorphism mediates allele-specific transcription via Poly(ADP-Ribose) polymerase 1 in macrophages engulfing apoptotic cells. J Immunol 184:3718–24. 10.4049/jimmunol.0903613 20181890 10.4049/jimmunol.0903613 PMC3637664

[21] Barber DL, Wherry EJ, Masopust D, et al (2006) Restoring function in exhausted CD8 T cells during chronic viral infection. Nature 439:682–7. Retrieved from: https://pubmed.ncbi.nlm.nih.gov/16382236/. Cited 2024 10.1038/nature04444 16382236

[22] Zhang T, Yu-Jing L, Ma TFront Immunol Frontiers Media S.A.,

[23] Huff V (2011) Wilms’ tumours: about tumour suppressor genes, an oncogene and a chameleon gene. Nat Rev Cancer 11(2):111 21248786 10.1038/nrc3002 PMC4332715

[24] Hastie ND: Wilms’ tumour 1 (WT1) in development, homeostasis and disease. Development (Cambridge) Company of Biologists Ltd, 144:2862–72, 2017 10.1242/dev.153163 28811308

[25] Harris PA, Taylor R, Thielke R, et al (2009) Research Electronic Data Capture (REDCap) - a metadata-driven methodology and workflow process for providing translational research informatics support. J Biomed Inform NIH Public Access, 42:377. Retrieved from: /pmc/articles/PMC2700030/. Cited 2024 10.1016/j.jbi.2008.08.010 PMC2700030 18929686

[26] Harris PA, Taylor R, Minor BL, et al (2019) The REDCap Consortium: Building an International Community of Software Platform Partners. J Biomed Inform NIH Public Access, 95:103208. Retrieved from: /pmc/articles/PMC7254481/. Cited 2024 10.1016/j.jbi.2019.103208 PMC7254481 31078660

[27] Livak KJ, Schmittgen TD (2001) Analysis of relative gene expression data using real-time quantitative PCR and the 2−ΔΔCT method. Methods 25:402–8. Retrieved from: https://linkinghub.elsevier.com/retrieve/pii/S1046202301912629. Cited 2022 10.1006/meth.2001.1262 11846609

[28] Fowler VG, O’Brien SM, Muhlbaier LH, et al: Clinical predictors of major infections after cardiac surgery. Circulation Lippincott Williams & Wilkins. https://pubmed.ncbi.nlm.nih.gov/16159846/. 10.1161/CIRCULATIONAHA.104.525790 16159846

[29] Aluru JS, Barsouk A, Saginala K, et al (2022) Valvular heart disease epidemiology. Med Sci 2022, Vol. 10, Page 32 Multidisciplinary Digital Publishing Institute, 10:32. Retrieved from: https://www.mdpi.com/2076-3271/10/2/32/htm. Cited 2025

[30] Cohen G (2020) Immune Dysfunction in Uremia 2020. Toxins 2020, Vol. 12, Page 439 Multidisciplinary Digital Publishing Institute, 12:439. Retrieved from: https://www.mdpi.com/2072-6651/12/7/439/htm. Cited 2025

[31] Lee TH, Chen JJ, Wu CY, et al: Immunosenescence, gut dysbiosis, and chronic kidney disease: Interplay and implications for clinical management. Biomed J Elsevier B.V., 472024 [cited 2025]Retrieved from: https://pubmed.ncbi.nlm.nih.gov/37524304/10.1016/j.bj.2023.100638 PMC10979181 37524304

[32] Kato S, Chmielewski M, Honda H, et al: Aspects of immune dysfunction in end-stage renal disease. Clinical Journal of the American Society of Nephrology Clin J Am Soc Nephrol, 3:1526–33, 2008 [cited 2025]Retrieved from: https://pubmed.ncbi.nlm.nih.gov/18701615/10.2215/CJN.00950208 PMC4571158 18701615

[33] Betjes MGH: Immune cell dysfunction and inflammation in end-stage renal disease. Nat Rev Nephrol Nat Rev Nephrol, 9:255–65, 2013 [cited 2025]Retrieved from: https://pubmed.ncbi.nlm.nih.gov/23507826/10.1038/nrneph.2013.44 23507826

[34] Kaul H, Girndt M, Sester U, et al: Initiation of hemodialysis treatment leads to improvement of T-cell activation in patients with end-stage renal disease. Am J Kidney Dis W.B. Saunders, 35:611–6, 2000 [cited 2025]Retrieved from: https://www.sciencedirect.com/science/article/abs/pii/S0272638600700060 10.1016/s0272-6386(00)70006-0 10739780

[35] Peroumal D, Jawale C V., Choi W, et al: The survival of B cells is compromised in kidney disease. Nature Communications 2024 15:1 Nature Publishing Group, 15:1–19, 2024 [cited 2025]Retrieved from: https://www.nature.com/articles/s41467-024-55187-w 10.1038/s41467-024-55187-w PMC11685736 39738044

[36] Colombo G, Astori E, Landoni L et al (2022) Effects of the uremic toxin indoxyl sulphate on human microvascular endothelial cells. J Appl Toxicol 42:1948–61. 10.1002/jat.4366 35854198 10.1002/jat.4366 PMC9796800

[37] Bhargava S, Merckelbach E, Noels H, et al: Homeostasis in the Gut Microbiota in Chronic Kidney Disease. Toxins 2022, Vol. 14, Page 648 Multidisciplinary Digital Publishing Institute, 14:648, 2022 [cited 2025]Retrieved from: https://www.mdpi.com/2072-6651/14/10/648/htm 10.3390/toxins14100648 PMC9610479 36287917

[38] Huo A, Wang F (2024) Berberine alleviates ischemia reperfusion injury induced AKI by regulation of intestinal microbiota and reducing intestinal inflammation. BMC Complement Med Ther BioMed Central Ltd, 24:1–12. Retrieved from: https://link.springer.com/articles/10.1186/s12906-023-04323-y. Cited 2025 10.1186/s12906-023-04323-y PMC10826000 38291383

[39] Locham S, Naazie I, Canner J, et al (2021) Incidence and risk factors of sepsis in hemodialysis patients in the United States. J Vasc Surg Mosby 73:1016–1021.e3. Retrieved from: https://www.sciencedirect.com/science/article/pii/S0741521420316980. Cited 2025 10.1016/j.jvs.2020.06.126 32707386

[40] Saraiva M, O’Garra A (2010) The regulation of IL-10 production by immune cells. Nat Rev Immunol 10:3. Nature Publishing Group, 10:170–81, 2010. Retrieved from: https://www.nature.com/articles/nri2711. Cited 2025. 10.1038/nri2711 20154735

[41] Van Der Poll T, Van De Veerdonk FL, Scicluna BP, et al (2017) The immunopathology of sepsis and potential therapeutic targets. Nature Reviews Immunology 2017 17:7. Nature Publishing Group, 17:407–20. Retrieved from: https://www.nature.com/articles/nri.2017.36. Cited 2025 10.1038/nri.2017.36 28436424

[42] Goldmann O, Nwofor OV, Chen Q, et al: Mechanisms underlying immunosuppression by regulatory cells. Front Immunol Front Media SA, 152024. Retrieved from: https://www.frontiersin.org/articles/10.3389/fimmu.2024.1328193/full 10.3389/fimmu.2024.1328193 PMC10876998 38380317

[43] Arellano-Rodriguez M, Zapata-Benavides P, Arellano-Rodriguez NC, et al (2021) The inflammatory process modulates the expression and localization of WT1 in podocytes leading to kidney damage. In Vivo (Brooklyn) International Institute of Anticancer Research, 35:3137. Retrieved from: https://pmc.ncbi.nlm.nih.gov/articles/PMC8627738/. Cited 2025 10.21873/invivo.12608 PMC8627738 34697144

