# Correction to: Gene expression kinetics in Sepsis After Cardiac Surgery (SACS): a multicentric prospective observational study

**DOI:** 10.1186/s44158-025-00297-0

**Published:** 2025-11-11

**Authors:** Rosa Paola Radice, Giuseppe Martelli, Mauro D’Amora, Pierpaolo Dambruoso, Domenico Paparella, Raffaele Mandarano, Giuseppe Olivo, Massimo Scolaro, Domenico Sarubbi, Alessandro Strumia, Maria Calabrese, Andrea Scapigliati, Francesco Greco, Mary Nardi, Stefano Beccaria, Andrea Costamagna, Luca Brazzi, SIAARTI Study Group, Domenico Abelardo, Pasquale Raimondo, Gianluca Paternoster

**Affiliations:** 1 Department of Science, University of Basilicata, Via Dell’ ateneo lucano 10, Potenza, PZ 85100 Italy; 2 AlgaeBioMed srl, via Luigi Kossuth 7, Rome, RM 00149 Italy; 3 AOR San Carlo - Via Potito Petrone, Potenza, PZ 85100 Italy; 4 Cardiovascular Anesthesia and Intensive Care Unit Ospedale Privato, Accreditato Santa Maria - GVM Care & Research, Bari, Italy; 5 Cardiac Anaesthesia and ICU, Department of Anaestesiology, Careggi University Hospital, Largo Giovanni Alessandro Brambilla, 3, Florence, FI 50134 Italy; 6 Unit of Cardiac Anesthesia and Intensive Care, Fondazione Toscana Gabriele Monasterio, Hospital of Massa, Pisa, Italy; 7 U.O.C. Di Anestesia E Rianimazione ‑ Policlinico Campus Bio Medico Di Roma Scuola Di Specializzazione in Anestesia E Rianimazione, Universita Campus Bio Medico Di Roma Via Alvaro del Portillo, Rome, 200 ‑ 00128 Italy; 8 Department of Cardiovascular Sciences, Intensive Care Unit, Fondazione Policlinico Universitario “A. Gemelli” IRCCS, Rome, Italy; 9 Fondazione Di Religione E Di Culto, Casa Sollievo Della Sofferenza, San Giovanni Rotondo, FG Italy; 10 Cardiovascular Anaesthesia and Intensive Care, Mauriziano Hospital, Turin, Italy; 11 Dipartimento Di Scienze Chirurgiche, Universita Degli Studi Di Torino, A.O.U Citta della Salute e della Scienza di Torino, Turin, Italy; 12 Department of Medical and Surgical Sciences, Italy. Regional Epilepsy Centre, Magna Graecia University of Catanzaro, Great Metropolitan “Bianchi-Melacrino-Morelli” Hospital, Reggio Calabria, Italy; 13 Department of Precision‑Regenerative Medicine and Jonic Area (DiMePRe‑J), Section of Anesthesiology and Intensive Care Medicine, University of Bari “Aldo Moro”, Bari, Italy

**Correction: J Anesth Analg Crit Care 5, 57 (2025)**

**https://doi.org/10.1186/s44158-025–00277−4**

The original publication of this article contained 2 errors:

- Several bibliographic references in the abstract were included
- The data tabulation in table 1

The incorrect and correct information is shown in this correction article. The original article has been updated.

**Table:** 

Incorrect Abstract	Correct abstract
**Objectives** Surviving Sepsis Campaign (SSC) defined Sepsis as “life-threatening organ dysfunction caused by a dysregulated host response to infection” (De Backer D et al, Crit Care Med, n.d.). Sepsis remains one of the leading causes of morbidity and mortality (17–65% (De Oliveira DC, Arq Bras Cardiol Sociedade Brasileira de Cardiologia - SBC 94:352–6, 2010)) worldwide and it still remains a challenge to be defined and for which an appropriate treatment is desired (Chiu and Legrand, Curr Opin Anaesthesiol 34:71–6, 2021). Different studies have been conducted on genes coding for inflammatory cytokines whose could predispose to the development of sepsis [e.g., IL-10 PD1 and WT1] (Gupta DL et al, Infectious Process Sepsis, 202). **Design** This multicentric observational prospective study aims to evaluate blinding the genetic expression kinetics of different molecules involved in the inflammatory process, IL10, PD1 and WT1, to search for a possible molecular predictive marker of sepsis. **Setting** Nine University teaching Hospitals in Italy take part in this study in collaboration with the Department of Applied Science (DISBA) of the University of Basilicata. **Participants** One hundred sixty-two patients, under elective cardiac and on pump surgery were enrolled in the study. **Interventions** From each patient 4 blood samples were collected during and at the end of the surgery, following the study design. **Measurements and main results** We observed, 30 min after the start of the surgery, lower gene expression levels of IL10 and PD1 in septic patients compared to non-septic (*p* < 0.05), but considering all the timepoint there are differences in gene expression modulation between the groups. **Conclusion** These results confirmed the dysregulated immune response in septic patients compared to non-septic, highlight how a measurement of the gene expression could help to optimize procedures and pay attention to more susceptible patients.	**Objectives** Surviving Sepsis Campaign (SSC) defined Sepsis as “*life-threatening organ dysfunction caused by a dysregulated host response to infection*”. Sepsis remains one of the leading causes of morbidity and mortality (17–65 %) worldwide and it still remains a challenge to be defined and for which an appropriate treatment is desired. Different studies have been conducted on genes coding for inflammatory cytokines whose could predispose to the development of sepsis [e.g., IL-10 PD1 and WT1]. **Design** This multicentric observational prospective study aims to evaluate blinding the genetic expression kinetics of different molecules involved in the inflammatory process, IL10, PD1 and WT1, to search for a possible molecular predictive marker of sepsis. **Setting** Nine University teaching Hospitals in Italy take part in this study in collaboration with the Department of Applied Science (DISBA) of the University of Basilicata. **Participants** One hundred sixty-two patients, under elective cardiac and on pump surgery were enrolled in the study. **Interventions** From each patient 4 blood samples were collected during and at the end of the surgery, following the study design. **Measurements and Main Results** We observed, 30 minutes after the start of the surgery, lower gene expression levels of IL10 and PD1 in septic patients compared to non-septic (p<0.05), but considering all the timepoint there are differences in gene expression modulation between the groups. **Conclusion** These results confirmed the dysregulated immune response in septic patients compared to non-septic, highlight how a measurement of the gene expression could help to optimize procedures and pay attention to more susceptible patients.

Incorrect table 1

**Table:** 

**Inclusion criteria**	**Elective cardiac surgery**
	On pump surgery
	Age ≥ 18 years
Exclusion criteria	Emergency surgery
	Active endocarditis
	Previous sepsis or septic shock
	Unsigned informed consent
	Age < 18 years

Correct table 1

**Table:** 

**Inclusion criteria**	Elective cardiac surgery
	On pump surgery
	Age ≥18 years
**Exclusion criteria**	Emergency surgery
	Off pump surgery
	Active endocarditis
	Previous sepsis or septic shock
	Unsigned informed consent
	Age <18 years

